# Emerging Trends of Self-Harm Using Sodium Nitrite in an Online Suicide Community: Observational Study Using Natural Language Processing Analysis

**DOI:** 10.2196/53730

**Published:** 2024-05-02

**Authors:** Sudeshna Das, Drew Walker, Swati Rajwal, Sahithi Lakamana, Steven A Sumner, Karin A Mack, Wojciech Kaczkowski, Abeed Sarker

**Affiliations:** 1 Department of Biomedical Informatics, School of Medicine, Emory University, Atlanta, GA, United States; 2 Department of Behavioral, Social, and Health Education Sciences, Rollins School of Public Health, Emory University, Atlanta, GA, United States; 3 Department of Computer Science and Informatics, Emory University, Atlanta, GA, United States; 4 National Center for Injury Prevention and Control, Centers for Disease Control and Prevention, Atlanta, GA, United States; 5 Department of Biomedical Engineering, Georgia Institute of Technology and Emory University, Atlanta, GA, United States

**Keywords:** online suicide community, suicide, sodium nitrite, sodium nitrite sources, mental health, adolescent, juvenile, self harm, Sanctioned Suicide, online forum, US, public health, surveillance, data mining, natural language processing, machine learning, usage, suicidal, accuracy, consumption, information, United States

## Abstract

**Background:**

There is growing concern around the use of sodium nitrite (SN) as an emerging means of suicide, particularly among younger people. Given the limited information on the topic from traditional public health surveillance sources, we studied posts made to an online suicide discussion forum, “Sanctioned Suicide,” which is a primary source of information on the use and procurement of SN.

**Objective:**

This study aims to determine the trends in SN purchase and use, as obtained via data mining from subscriber posts on the forum. We also aim to determine the substances and topics commonly co-occurring with SN, as well as the geographical distribution of users and sources of SN.

**Methods:**

We collected all publicly available from the site’s inception in March 2018 to October 2022. Using data-driven methods, including natural language processing and machine learning, we analyzed the trends in SN mentions over time, including the locations of SN consumers and the sources from which SN is procured. We developed a transformer-based source and location classifier to determine the geographical distribution of the sources of SN.

**Results:**

Posts pertaining to SN show a rise in popularity, and there were statistically significant correlations between real-life use of SN and suicidal intent when compared to data from the Centers for Disease Control and Prevention (CDC) Wide-Ranging Online Data for Epidemiologic Research (⍴=0.727; *P*<.001) and the National Poison Data System (⍴=0.866; *P*=.001). We observed frequent co-mentions of antiemetics, benzodiazepines, and acid regulators with SN. Our proposed machine learning–based source and location classifier can detect potential sources of SN with an accuracy of 72.92% and showed consumption in the United States and elsewhere.

**Conclusions:**

Vital information about SN and other emerging mechanisms of suicide can be obtained from online forums.

## Introduction

### Background

Suicide rates in the United States continue to rise and remain near their highest levels in more than 2 decades [1,2]. There were 48,183 suicides in the United States in 2021, which is 5% higher than the reported number in 2020 [3]. Poisoning is the most common mechanism of suicide attempts in the United States [4] and the third-leading mechanism involved in suicides [5]. One factor complicating suicide prevention efforts is the continual emergence and promotion of new means by which one can attempt suicide, such as novel substances.

Since 2019, a growing trend of using sodium nitrite (SN), a common food additive, for self-harm has been reported [6,7]. SN has traditionally been used as a food preservative and coloring agent, in addition to use as a corrosion inhibitor. As such, it is widely available for purchase. An alarming development has been the sale of “suicide kits” in online marketplaces, which comprise SN in addition to instructional material on attempting suicide [8]. Instances of the use of such suicide kits have been reported in the literature [9]. Furthermore, media reports of celebrity suicides from SN ingestion have raised public awareness of this means of self-harm. As a widely available, water-soluble salt with reported lethal dosages of as low as 0.7 g [10], the potentially growing popularity of SN as a suicide mechanism is concerning. Between 2018 and 2020, the annual suicide rate involving SN increased from 0.01 to 0.09 per 100,000 person-years in the United States [11]. Although there are concerns about increased youth suicides due to media contagion [12], studies show that adhering to suicide reporting guidelines [13] can raise awareness and have a protective effect through the coverage of positive coping mechanisms [14].

The consumption of SN induces methemoglobinemia, a condition resulting in hypoxia, and if not treated promptly, it can result in death. Although the unintentional consumption of SN due to misleading or dubious storage practices is of concern [15], the consumption of SN with the intent of self-harm has also been reported in countries such as Australia, Portugal, and South Korea [6]. It is believed that global popularization of SN, instruction on its use in suicide, and sharing of information about procuring SN has been facilitated by online forums such as “Sanctioned Suicide” [15], about which little is known.

### Online Suicide Forums

Online forums provide a platform for users with similar interests to share their views on common topics of interest. Internet support forums exist for a wide variety of health-related issues, including mental health and suicide-related behaviors. Sanctioned Suicide is the successor of the eponymous subreddit (a topic-specific forum), which was banned in March 2018 for violating Reddit’s policies on content promoting self-harm and specific suicide methods [15]. The purpose of the website, as mentioned in their frequently asked questions [16], is to allow individuals to discuss suicide—including suicide methods—without the content screening that occurs on more prominent social media platforms. Thus, this forum encapsulates a large amount of suicide-related information that can be of high utility for planning and enacting public health measures to prevent suicides. The large volume of data, however, also makes it impractical to manually review the content continuously to generate timely and evolving insights. Automated methods are thus required to optimally leverage this resource of publicly available information.

### Objective

Although the use of SN for suicide has elevated to the level of congressional interest in the United States [17], little is known about epidemiologic trends from Sanctioned Suicide that could inform prevention efforts. The key strategies highlighted in the *Suicide Prevention Resource for Action* [18] by the US Centers for Disease Control and Prevention (CDC) call for “data-driven strategic planning with engagement from multi-sectoral partners” for making decisions associated with the prevention of suicides in the United States [17]. In line with the objectives and guidance set out in this resource, in this paper, we adopt a data-driven approach using natural language processing (NLP) and other techniques to study large-scale public posts from Sanctioned Suicide to answer critical questions relevant to suicide prevention efforts:

1. Does interest in SN appear to be increasing over time on the forum, and how does this interest compare to other mechanisms of suicide?
2. Are there other co-occurring substances or topics of interest relevant to SN?
3. What are the leading countries and vendors of SN that are being promoted?

## Methods

### Data Collection and Preprocessing

**Figure 1.**
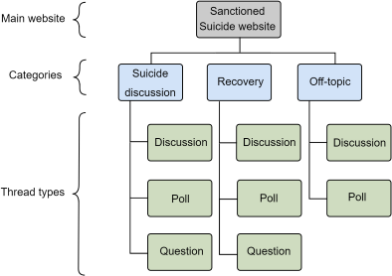
Overall structure of the Sanctioned Suicide website. Threads are organized under 3 broad categories: suicide discussion, recovery, and off-topic.

We collected data from the website “Sanctioned Suicide,” an online community dedicated to discussing “the topic of suicide without the censorship of other places” [16], which has received substantial attention because of its rising popularity in the recent past [15]. Figure 1 presents the structure of the Sanctioned Suicide network. As the figure illustrates, the social network is broadly divided into 3 types of discussion based on topic (*suicide*, *recovery*, and *off-topic*). We collected all posts available on the website from March 22, 2018 (the date on which the website went live), to October 7, 2022 (the date of data collection). We removed duplicate posts and applied preprocessing steps that are standard in NLP, namely tokenization, lowercasing, punctuation removal, stop-word removal, and lemmatization.

### Data-Driven Analysis of Suicide Mechanisms

#### Suicide Mechanism Detection and Trend Analysis

Our first objective was to detect mentions of SN as well as other specific substances and suicide methods. As is common over social media, many lexical variants are used to discuss SN and other substances or methods. Not including commonly used lexical variants leads to low-sensitivity data collection [19]. We took a data-driven approach to identify all the relevant lexical variants for SN. We trained a Word2Vec model using n-grams (n=1, 2, and 3) from the entire data set, which enabled us to automatically identify lexical expressions that are the most semantically similar to SN. We manually reviewed these terms and identified, in addition to alternative names for SN, keywords associated with other suicide mechanisms. In consultation with our subject matter experts, we manually grouped these keywords into 14 categories: “sodium nitrite,” “cyanides,” “firearms,” “hanging,” “acid regulators,” “ricin,” “plant-based poisons,” “antiemetics,” “other preservatives,” “nitric oxide,” “household chemicals,” “barbiturates,” “benzodiazepines,” and “opioids.” The complete list of suicide mechanism–related terms identified is given in Table S1 in Multimedia Appendix 1.

We performed automatic searches over the whole data set to compute the frequencies of posts mentioning each method of suicide over time. A post that mentioned any number of lexical variants associated with a suicide method counted toward that method. For example, a post mentioning only “NaNO_2_” is assigned the group “sodium nitrite,” whereas a post mentioning “gun” and “full suspension” is assigned the group labels “firearms” and “hanging*.*” We computed the monthly and normalized frequencies of posts mentioning each of these categories to analyze their temporal trends.

#### Comparison of Trends With Traditional Data

We compared the temporal trends of SN mentions we discovered from the above analysis with relevant metrics reported in two more traditional sources: (1) intentional exposures to SN in the US National Poison Data System (NPDS) and (2) death counts in the CDC Wide-Ranging Online Data for Epidemiologic Research (WONDER) database. For the first comparison, we compared our data against the quarterly intentional exposures to SN from the NPDS reported by McCann et al [10]. For the second comparison, we compared against the reported deaths under the underlying cause of death codes U03, X60–X84, and Y87.0 and multiple causes of death code T50.6 from the CDC WONDER database. For the latter, to make the comparison better aligned with the traditional data source, we combined our keyword mention counts pertaining to “sodium nitrite” and “other preservatives.” We performed a Spearman rank correlation test to assess possible associations between the pairs of statistics.

#### Co-Occurrence Analysis and Topic Modeling

We performed a co-occurrence analysis to compute the number of times different suicide methods we already identified were mentioned together. The intuition behind this analysis was that suicide methods that are considered together or substances that are taken together (eg, substances taken alongside SN) are likely to be mentioned more frequently in the same posts.

To obtain further insights about the topics associated with SN chatter, we conducted a topic modeling experiment. Topic modeling is a class of unsupervised algorithms that attempt to identify clusters of lexical elements that belong to latent topics from large sets of texts. Since the process is purely unsupervised, the topic clusters are not known a priori. We applied the BERTopic model, an unsupervised topic modeling approach that automatically clusters content from posts into a mathematically optimized number of topics [20].

### Identification of SN Sources and Consumer Locations

#### Named Entity Recognition

**Figure 2.**
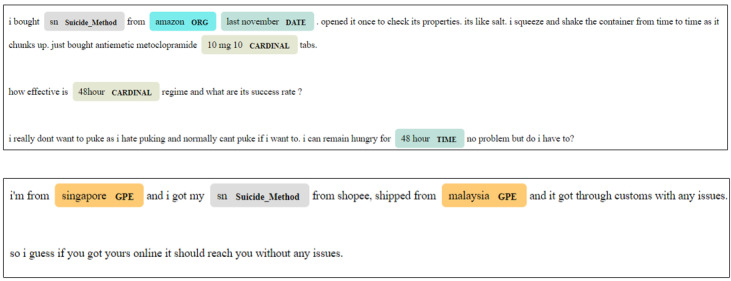
Examples of named entity recognition from Sanctioned Suicide posts from March 2018 to September 2022. Detected entities, their spans, and their inferred types are shown. GPE: geopolitical entity; ORG: organization; SN: sodium nitrite.

Purchasing, sourcing, and procurement of SN were identified as common topics of discussion during the analyses mentioned above (see the *Results* section), so we used a 2-fold approach to identify information about both the retail venues and the geographic locations where SN was being sought for purchase. Specifically, we leveraged named entity recognition (NER) models trained on large web-based data sets to identify potential sources of SN: locations, organizations, and keywords used to look for SN on online marketplaces. NER is an information extraction technique used to discover named entities (such as organizations, locations, etc) in a textual corpus (Figure 2).

We used an NER algorithm available in the Python programming language (spaCy NER [21]) to detect possible locations or sources from which people seek or obtain SN. Apart from using spaCy’s location entity label, we created a custom entity called “Suicide_Method” that would identify the substance in the text and highlight it. We used the rule-based pattern recognizer in spaCy to detect mentions of SN. In particular, we used the pattern “sn” to detect mentions of SN (the code snippet is shown in Multimedia Appendix 1). Posts where SN was mentioned in conjunction with location names were used for further analysis. The location entity recognizer of spaCy has a reported *F*_1_-score of 0.916 [22].

#### Location Mention–Intent Classifier

The locations identified by the NER methods represented both sources of SN and consumer locations, and thus, the process required further disambiguation. We modeled this disambiguation as a supervised classification task and trained a transformer-based location classifier to classify the locations obtained from NER as “consumer location” and “purchase location.” Transformer models leverage large amounts of “pre-trained” language data, which can then be fine-tuned on a specific task—such as location type identification, in the case of this study [23]. Sentence-level annotation of location mentions was carried out by 2 of the authors to create a gold-standard training data set for fine-tuning the model. The annotation process was carried out iteratively, with the annotation guidelines being refined after each round of annotation. Disagreements were resolved after a detailed discussion between 3 of the authors to reach a consensus after each round. Two rounds of annotation were performed, and interannotator agreement was computed based on Cohen κ, revealing good agreement for both source location (κ=0.80) and consumer location (κ=0.84) [24]. A total of 722 samples were annotated, of which 577 (80%) were used for training and the remaining 145 (20%) were used for testing.

From the many transformer-based models that are publicly available, we chose the RoBERTa model [25], which is based on Bidirectional Encoder Representations From Transformers [26] and has been shown to achieve state-of-the-art results on several language processing tasks similar to those by the base model, including for health-related, social media–based text classification tasks [27]. Our use of “state-of-the-art” refers to the best-performing machine or deep learning models currently available for each task, in the rest of the paper. We used the embeddings obtained from RoBERTa to fine-tune our model using the training data. We computed the distributions of the sources and locations identified automatically for analysis. Classification results and all outputs are provided in the *Results* section.

### Ethical Considerations

This study was deemed to be exempt from review (publicly available data) by the Emory University Institutional Review Board. Our analyses use publicly available, user-generated content from an online forum where users remain anonymous by default. We do not use any personally identifiable information and only report aggregated data.

## Results

### Data Collection and Frequency Analysis

#### Overview

A total of 1,337,982 posts were collected. Of these, 1,302,620 were posted under the “Discussion” threads; 28,666 under the “Poll” thread; and 6696 under the “Question” threads. Preprocessing and removal of duplicate posts resulted in 1,329,042 total posts. There was a steady increase in the total number of posts from 2018 to 2020, followed by a slight decrease in 2021. The highest number of posts on the website was made in 2020. In the months leading up to September 2022, the final full month of data collection, the total number of monthly posts was generally higher than in the corresponding months in 2021 (Figure S1 in Multimedia Appendix 1).

#### Temporal SN Trends

**Figure 3.**
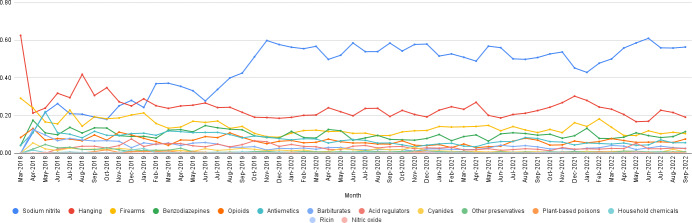
Normalized frequencies of posts on Sanctioned Suicide mentioning potential suicide means and related substances per month from March 2018 to September 2022.

Figure 3 shows the relative monthly frequencies for posts mentioning SN, as well as other substances and methods associated with suicides. From the figure, it can be observed that SN was the most popular means of suicide discussed in this online community, and the frequency of mentions of SN increased over time. A sharp rise in SN mentions can be seen towards the end of 2019, with the frequency of mentions remaining elevated thereafter.

#### Comparison of Trends With Traditional Data

**Figure 4.**
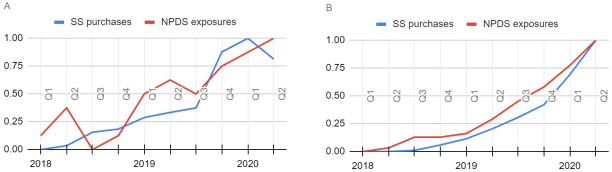
Comparison of normalized frequencies of purchase mentions on Sanctioned Suicide (SS) versus National Poison Data System (NPDS) exposures as reported in McCann et al [10], from 2018 to 2020. (A) Noncumulative frequencies; (B) cumulative frequencies. Q: quarter.

SN-related deaths due to intentional consumption have increased since 2019 [28]. Our analyses align with this uptick of SN ingestions and suicides: a sharp increase in the mentions of purchases of SN was seen toward the end of 2019 (Figure 3). In our comparison against the NPDS data, we obtained a Spearman ⍴ of 0.866 (*P*=.001), revealing a statistically significant association between the 2 data sources. On visualizing the normalized frequencies of intentional exposures in the NPDS and purchases made as obtained from our data set (see the *Sources and Consumer Locations* section), we found that both the noncumulative and cumulative frequencies showed similar trends (Figure 4) for the 10 quarters spanning from 2018 to 2020 for which data from both sources were available (quarters 1 to 4 for 2018 and 2019, and quarters 1 and 2 for 2020). Although the mentions of purchases on Sanctioned Suicide are not exclusive to the United States, the similar trends are a strong indicator of online suicide community content reflecting real-life suicide incidences.

**Figure 5.**
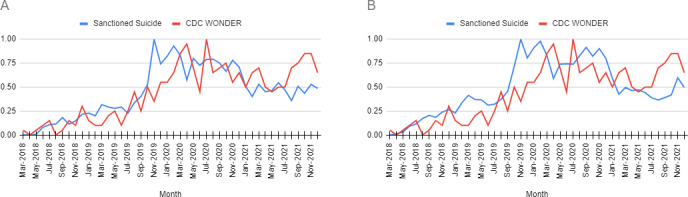
Comparison of normalized frequencies. (A) Purchase mentions of “sodium nitrite“ on Sanctioned Suicide versus actual deaths reported in the CDC WONDER database from 2018 to 2021; (B) keyword mentions of “sodium nitrite” and “other preservatives” on Sanctioned Suicide versus actual deaths reported in the CDC WONDER database. Suicide deaths involving chelating agents were identified by using the *International Classification of Diseases, Tenth Revision* underlying cause of death codes U03, X60–X84, and Y87.0 and multiple causes of death code T50.6. CDC: Centers for Disease Control and Prevention; WONDER: Wide-Ranging Online Data for Epidemiologic Research.

In the comparison against metrics from the CDC WONDER database, we obtained a Spearman ⍴ of 0.727 (*P*<.001) for month-by-month frequencies during the time period from March 2018 to December 2021 for SN purchases versus actual deaths and a Spearman ⍴ of 0.775 (*P*<.001) for keyword mentions versus actual deaths, revealing statistically significant correlation in both cases (Figure 5).

#### Co-mentioned Suicide Mechanisms

**Figure 6.**
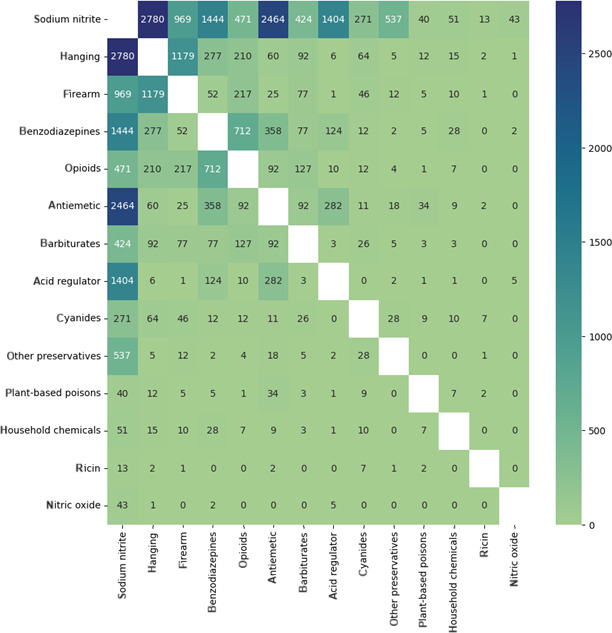
Heatmap illustrating the most commonly co-occurring suicide mechanism mentions in Sanctioned Suicide posts from March 2018 to September 2022.

The heatmap in Figure 6 presents the co-occurrence frequencies between different substances and methods. As illustrated in the figure, co-occurring mentions of SN and antiemetics are common. Manual qualitative inspection of these posts revealed that antiemetics are often mentioned when discussing SN, as forum users recommended these substances to ensure that individuals do not feel nauseous after consuming SN.

#### Topic Modeling

Topic modeling revealed further insights and some key differences in the content of posts. First, the topics discovered reinforced some of the insights revealed in Figure 3. For the SN group, unigram and bigram topic clusters represented the following:

- Substances that are potentially coingested (benzodiazepines, antiemetics, and metoclopramide);
- Dosage amounts (“tablespoon” and “grams”);
- Sourcing-related questions and information (“I’m looking,” “source,” “ordered,” and “package”);
- Comparison with other mechanisms of suicide (“hanging” and “shotgun”);
- Mechanism of action and symptoms (“hypoxia” and “peaceful way”); and
- Descriptions of experiences, thoughts, and suicide notes (“failed attempt,” “feel like,” and “I’m sorry”).

Figures S2 and S3 in Multimedia Appendix 1 present all the topics.

### Sources and Consumer Locations

#### Overview

**Figure 7.**
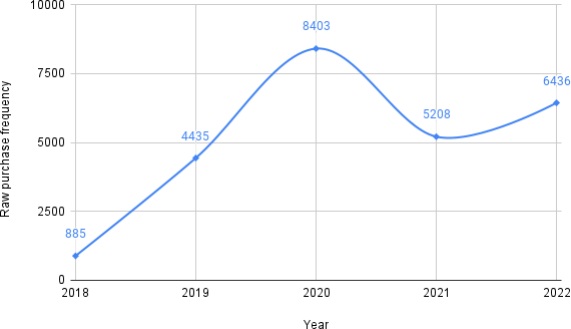
Raw yearly purchase frequency of sodium nitrite from Sanctioned Suicide posts from March 2018 to December 2022 (extrapolated to the period from October to December 2022).

**Figure 8.**
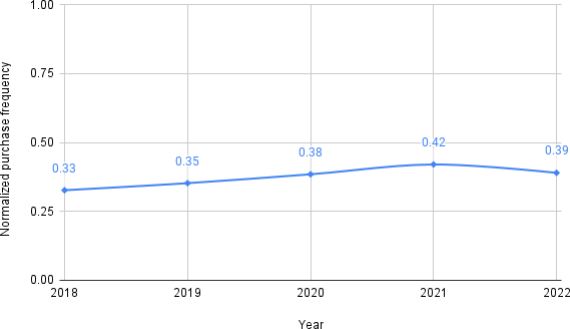
Normalized yearly purchase frequency of sodium nitrate from Sanctioned Suicide posts from March 2018 to December 2022 (extrapolated to the period from October to December 2022).

Based on the aforementioned topic modeling experiment results and supplemented with manual qualitative inspection of SN-related posts, we curated a list of phrases related to sourcing (eg, “seller,” “bought,” and “purchase”). The complete list is given in Table S2 in Multimedia Appendix 1. Since we collected data from Sanctioned Suicide on October 7, 2022, we extrapolated the frequency of posts from 279 days before (January 1, 2022, to October 7, 2022) by multiplying the per-day frequency with the total number of days in the year. Frequency analysis of sourcing-related posts pertaining to SN shows a sharp rise in “purchase” toward the end of 2019, with the highest raw annual “purchase” frequency observed in 2020 (Figure 7). Figure 8 shows the sourcing frequency (purchase frequency) of SN normalized by the posting frequency of SN-related topics. We found that discussions about the sourcing of SN gradually increased over the years.

#### SN Sources and Consumer Locations

The NER approach detected locations that included countries, states, cities, counties, etc. We manually analyzed the detected locations to create a location-mapping dictionary, which was used to map cities to countries. The United States, the United Kingdom, Canada, China, and Germany were found to be the most popular potential locations for obtaining or using SN (Table 1). Since the locations detected were primarily from the United States, we also mapped locations detected from within the United States to the state level. The states of California, New York, Florida, Texas, and Oregon were found to be mentioned the most often in association with SN.

**Table 1.** Identified geographical locations from Sanctioned Suicide posts, from March 2018 to September 2022, associated with the sourcing or use of sodium nitrite.

Rank	Country-level locations		US state–level locations	
	Country	Frequency, n	US state	Frequency, n
1	United States	1043	California	149
2	United Kingdom	774	New York	104
3	Canada	358	Florida	67
4	China	202	Texas	63
5	Germany	185	Oregon	50
6	Mexico	170	Washington	41
7	Australia	164	Pennsylvania	30
8	India	163	North Carolina and Virginia^a^	23
9	Netherlands	146	Illinois	20
10	Switzerland	144	Arizona	18
11	Russia	140	Massachusetts	17
12	France	128	Utah	16
13	Japan and Spain^a^	96	Michigan and Nevada^a^	15
14	Poland	90	Colorado	13

a Locations with equal frequencies, presented in alphabetical order.

#### Location Mention–Intent Classification

Although NER-based geographical sources are informative, we found that consumers often tend to circumvent naming the geographical and online sources from where they obtained SN. Consider the following post: “I ordered it off of the big river in Brazil website.” NER identifies “Brazil” as a potential geographical source of SN in this example. However, the user is referring to the online source “Amazon” rather than the geographical source “Brazil.” Furthermore, locations mentioned in posts may also refer to the consumer’s location rather than the location where SN was sourced. This necessitated building a classifier to identify the intent of mentioning the location. Our proposed location mention–intent classifier achieved an accuracy of 72.92% on the unseen test data set, outperforming traditional machine learning–based baselines (Table 2).

**Table 2.** Performance of the transformer-based, location mention–intent classifier on Sanctioned Suicide posts from March 2018 to September 2022.

Model	Random classifier	Support vector machine classifier	Our model
Accuracy	0.25	0.34	0.72
Precision	0.25	0.37	0.73
Recall	0.25	0.32	0.76
*F*_1_-score	0.25	0.35	0.74

#### Sources and Consumer Locations

Based on the NER and subsequent classification process, the United States, the United Kingdom, Canada, Australia, and China were found to be the most popular source locations for obtaining SN (Table 3). The states of California, New Mexico, Florida, Rhode Island, and Oklahoma were reported to be the most popular sourcing locations for SN within the United States.

**Table 3.** Locations where sodium nitrite was potentially sourced from, as obtained from Sanctioned Suicide posts from March 2018 to September 2022.

Rank	Country-level locations		US state–level locations	
	Country	Frequency, n	State	Frequency, n
1	United States	586	California	55
2	United Kingdom	303	New Mexico	44
3	Canada	129	Florida	24
4	Australia	122	Rhode Island	21
5	China	83	Oklahoma	13
6	Germany and Mexico^a^	80	Virginia	12
7	India	67	Minnesota, New York, North Dakota, and Vermont^a^	8
8	Netherlands	53	Arizona, Georgia, Maryland, and Oregon^a^	7
9	Russia	45	Alabama, Illinois, Massachusetts, and Texas^a^	6

a Locations with equal frequencies, presented in alphabetical order.

The consumers who were interested in or attempted to obtain SN primarily were from the United States, the United Kingdom, Canada, Mexico, and Australia (Table 4). Within the United States, the states of California, New York, Texas, Florida, and Pennsylvania were found to be the most common locations of consumers attempting to obtain SN. For user-level post frequency, the mean was 72.63 (median 13, IQR 4-46; range 1-14,795).

**Table 4.** Locations where consumers attempting to procure sodium nitrite were from, as obtained from the Sanctioned Suicide posts from March 2018 to September 2022.

Rank	Country-level		US State-level	
	Country	Frequency, n	State	Frequency, n
1	United States	150	California	22
2	United Kingdom	107	New York and Texas^a^	10
3	Canada	41	Florida	6
4	Mexico	23	Pennsylvania	5
5	Australia	22	Oregon	4
6	China	21	Colorado, Maryland, Massachusetts, Oklahoma, and Washington^a^	3
7	India	15	Alaska, Illinois, New Jersey, Ohio, and Utah^a^	2
8	Russia and Switzerland^a^	12	Arizona, Connecticut, Georgia, Indiana, Kentucky, Louisiana, Michigan, Mississippi, South Carolina, and Wisconsin^a^	1
9	Brazil	10	N/A^b^	N/A

a Locations with equal frequencies, presented in alphabetical order.

b N/A: not applicable.

#### Potential Online Sources

Our NER-based approach also revealed potential online sources of obtaining SN or information about obtaining SN. Online marketplaces were the most commonly mentioned potential sources of SN (Table 5).

**Table 5.** Possible internet-based sources of sodium nitrite mentioned from Sanctioned Suicide posts from March 2018 to September 2022.

Rank	Online source	Frequency, n
1	Online	15,601
2	Google	6314
3	YouTube	5173
4	Amazon	2909
5	Facebook	2837
6	eBay	2373
7	Pharmacy	800
8	Walmart	621
9	Online pharmacy	593
10	Craigslist	148
11	Alibaba	84
12	Etsy	81
13	CVS	69
14	Tesco	51
15	Walgreens	45
16	AliExpress	26
17	Taobao	4

## Discussion

### Principal Findings

Our study is the first to conduct a comprehensive, NLP-based assessment of a large, popular, and public suicide forum. Our findings show that SN is the most popular method of suicide discussed on the forum, perhaps indicating the rising popularity of SN in real life. The trends we discovered suggest that the popularity of SN might still be increasing. Our study also revealed topics associated with SN discussions, among which sourcing was a common one. The application of automated NLP methods such as NER and classification enabled us to rapidly aggregate the locations and sources and compute their frequencies. Our findings and the data mining resources we are releasing with this paper will aid much-needed future research on this topic.

### Online Sources of SN

Our analyses show the distinct role of online marketplaces as a source of SN (Table 5). As a common food additive approved for use [29] in several countries, such as the United States [30], the United Kingdom [31], New Zealand, and Australia [32], SN is widely available for procurement. However, SN is now listed as a poison in the United Kingdom and, thus, is considered to be a reportable substance whose sale is regulated and requires an Explosive Precursors and Poisons license [31]. Some online marketplaces, such as Etsy and eBay [33], have now implemented restrictions prohibiting the sale of SN through their website [28]. Overall, however, there remains a wide availability of products containing SN on internet marketplaces.

### Trends in SN Use for Self-Harm

SN as a suicide mechanism has been reported in the medical literature as early as 1979 [34]. Since then, there have been a limited number of case reports in the literature through 2019, including one paper presenting 10 cases of SN consumption with the intent for self-harm [6]. Since 2019, case reports of SN-related deaths due to intentional consumption have increased [28], which is reflected in both the online community data as well as reported exposures (Figure 4) and deaths from official databases (Figure 5). This trend of rising instances of SN use for self-harm is concerning and may benefit from broader scientific interest. The literature on the topic is still sparse though, particularly at the intersection of SN and social media or internet-based data.

### Utility of Internet-Based Data

Internet-based data hold substantial potential for the surveillance of suicide methods, particularly emerging topics such as the one we studied in this paper. Recent advances in data-centric artificial intelligence methods, particularly NLP, have opened up opportunities for rapidly analyzing such data, as we did in this study. While our paper is the first to take such a data-driven approach to fully describe the contents of this forum from an epidemiologic perspective, other recent papers have attempted to analyze data from it. Sartori et al [35], for example, investigated the impact of COVID-19 by studying posts from this forum, and they found that COVID-19 appeared to be indirectly connected to causes of distress for the users, such as anxiety for the economy, but not directly to the growth of users on the forum. Dilkes [36] took a more linguistic investigation approach and analyzed changes in language to evaluate the social and psychological effect of participation in the forum. In a more recent commentary, Dinis-Oliveira and Durão [37] further highlighted the importance of studying the forum, how it plays a role in providing guidance on how to use SN as a means of suicide, and the rapid increase in its popularity. Although our study takes a necessary next step in the use of this data source for public health work, this information can also be leveraged to address this emerging public health problem in the United States and globally in the form of locally targeted interventions, such as notices for emergency personnel about signs, symptoms, and treatment in locations prone to the issue [38,39].

### Conclusion

In this study, we adopted a data-driven approach to analyze the trends in SN mentions on an online suicide forum using NLP and machine learning–based techniques. Our findings show that online forums can be an important source of information about emerging trends in suicide mechanisms. We also show that it is possible to obtain geographical trends of use and sourcing with our proposed location mention–intent classifier, with high accuracy. Since suicide is a rising concern in the United States and worldwide, we believe our study can be key to understanding temporal trends in suicide mechanisms and provide insights to the public health community by leveraging large amounts of online data sources.

### Limitations and Future Work

Since we used Sanctioned Suicide as our primary data source, a limitation of our study is that the distribution of users appears to be largely from the United States. This is reflected in both the location-based analyses and the close association of Sanctioned Suicide trends with the CDC WONDER and the NPDS data. Differences in access to the internet and cyber literacy across the world further contribute to this distribution. The posts used in this study were in English, which may also have caused users from non–English-speaking countries to be underrepresented. Our NLP and machine learning methods also impose some limitations—the classification abilities of our models are not perfect, and errors can affect downstream tasks. In our study, however, the presence of a large volume of data helps to offset the influence of individual errors; furthermore, the use of machine learning is necessary in analyzing data that are too large to qualitatively assess by manual processes.

In the future, we aim to attempt to improve our NER and classification approaches so that more accurate information about sources and locations can be obtained in close to real time. We will also attempt to develop NLP tools that can automatically discover novel substances and mechanisms gaining popularity in that community. The resources we are sharing with this publication (lexicons, lists of phrases and keywords, and language models) are intended to support researchers to conduct their own data-driven studies on the topic.

Intentional SN ingestion remains an ongoing concern for suicide prevention work. The ease of accessibility and lethality of the substance present a unique challenge for public health efforts. As nations consider both policy and programmatic options for enhanced prevention opportunities, the use of online data will remain critical to understand emerging trends in a timely fashion.

## Supplementary material

10.2196/53730 Multimedia Appendix 1 Full list of suicide mechanism keywords, posting frequency on Sanctioned Suicide, full list of sourcing-related keywords, sodium nitrate–related topics: unigrams and bigrams, and code snippet.

## Abbreviations

CDC: Centers for Disease Control and Prevention
NER: named entity recognition
NLP: natural language processing
NPDS: National Poison Data System
SN: sodium nitrite
WONDER: Wide-Ranging Online Data for Epidemiologic Research
