# Supplementary material for: Emerging Trends of Self-Harm Using Sodium Nitrite in an Online Suicide Community: Observational Study Using Natural Language Processing Analysis
Source: JMIR Ment Health. 2024 May 2;11:e53730. doi: 10.2196/53730 (PMC11085041; doi:10.2196/53730)
Supplement: Multimedia Appendix 1 [file mental-v11-e53730-s001.docx]

**Multimedia Appendix 1**

**Table S1.** Full list of suicide mechanism keywords used and their groupings.

| **Suicide Mechanism** | **Associated Keywords** |
| --- | --- |
| Sodium nitrite | sn, sni, sodium nitrite, sodium nitrate, nano3, nano2, snit, nitrite, nitrate, nitrit, nitrites, snwhat |
| acid regulator | acid reducer, acid regulator, anti acid, antacid, tagamet, tagmet, tagament, cimetidine |
| hanging | hanging, hang, full suspension, hanged |
| ricin | ricin |
| plant-based poisons | yew, datura, apocynaceae, pong pong, othalanga, cerberin |
| antiemetic | anti emetic, antiemetic |
| nitric oxide | nitric oxide |
| cyanides | potassium cyanide, sodium cyanide, cyanide, apple seeds, amygdalin, kcn, hcn, hydrogen cyanide |
| household chemicals | pesticide, drain cleaner, rat poison, butanediol |
| barbiturates | pentobarbital sodium, nembutal, sodium thiopental |
| other preservatives | azide, sodium azide, curing salts, curing salt, curing meat, meat curing |
| firearm | gun, firearm, firearms, handgun, shotgun, pistol, rifle, revolver, pistols, shotguns, guns, handguns, glock, magnum, shoot |
| benzodiazepines | alprazolam, clonazepam, diazepam, triazolam, lorazepam, midazolam, temazepam, oxazepam, nimetazepam, norflurazepam, metizolam, nitrazolam, adinazolam, phenazepam, pyrazolam, flualprazolam, flunitrazepam, flunitrazolam, bromazolam, nitrazepam, clonazolam, flubromazolam, bromazepam, etizolam, diclazepam, flubromazepam, benzos, benzodiazepine, benzo |
| opioids | hyrdrocodone, hydrocodiene, hydro codone, hydrocodene, hydrocordone, hydorcodone, hydrocodeine, hidrocodone, hyrocodone, hydrocone, hydrodone, hyrdocodone, hydrcodone, hydrocodones, hydrocordisone, hydocodone, hydrocode, hydrocodons, hydracodone, hydrocodone, hydrocodin, hydrocodine, hydrocodon, hydrocondone, hydrocodne, hydrocdone, hydros, 357s, lortab, lortabs, lorcet, lorcets, lortab, norco, vicoden, vicadin, viodin, vicodin, vicodines, vicodan, vicodien, viccodin, vocodin, vicondin, vicoding, vicodins, vicodon, vicidin, vidodin, vikodin, viacodin, vicodine, vicdin, vicotin, percoset, percocets, percocett, pecocets, percocoet, percocit, percet, percoct, percocet10, percicet, percocetes, percecet, percocet, oxocodone, oxycodene, oxycondone, oycodone, oxyxodone, oxycodones, oxicodone, oxy codone, oxycodone, oxycodine, roxycodone, ocycodone, oxycodons, oxcodone, oycondone, oxycodon, oyxcodone, oxcycodone, oxycodne, oxy, oxys, roxy, roxies, roxicodone, oc, percs, m30, m30s, dilaudid, hydromorphone, oxymorphone, ocycontin, oxcontin, oxcotin, oxcycontin, oxycotine, oxycontin, roxycontin, oycotin, oxyconton, oxycontine, oxycotins, oxycontin, oxycintin, oxy contin, oxicontin, oxycontins, oxycottin, oycontin, morphin, morfin, morphs, tramadol, trmadol, tramdol, tramadol, fentinyl, fentenyl, fenanyl, fentanly, fentnyal, fentanol, fental, fetanyl, fentayl, fentanayl, fentanyl, fentyl, fentanal, fetnyl, fentynyl, fentnayl, fentanl, fentyanl, fentonyl, fentanyal, fentany, fentnyl, fent, fents, carfentanil, carfentanyl, carfent, heroin, herroin, herioin, heroins, sufentanyl, sufentanil |

**Figure S1.** Posting frequency on Sanctioned Suicide, March 2018 to September 2022.


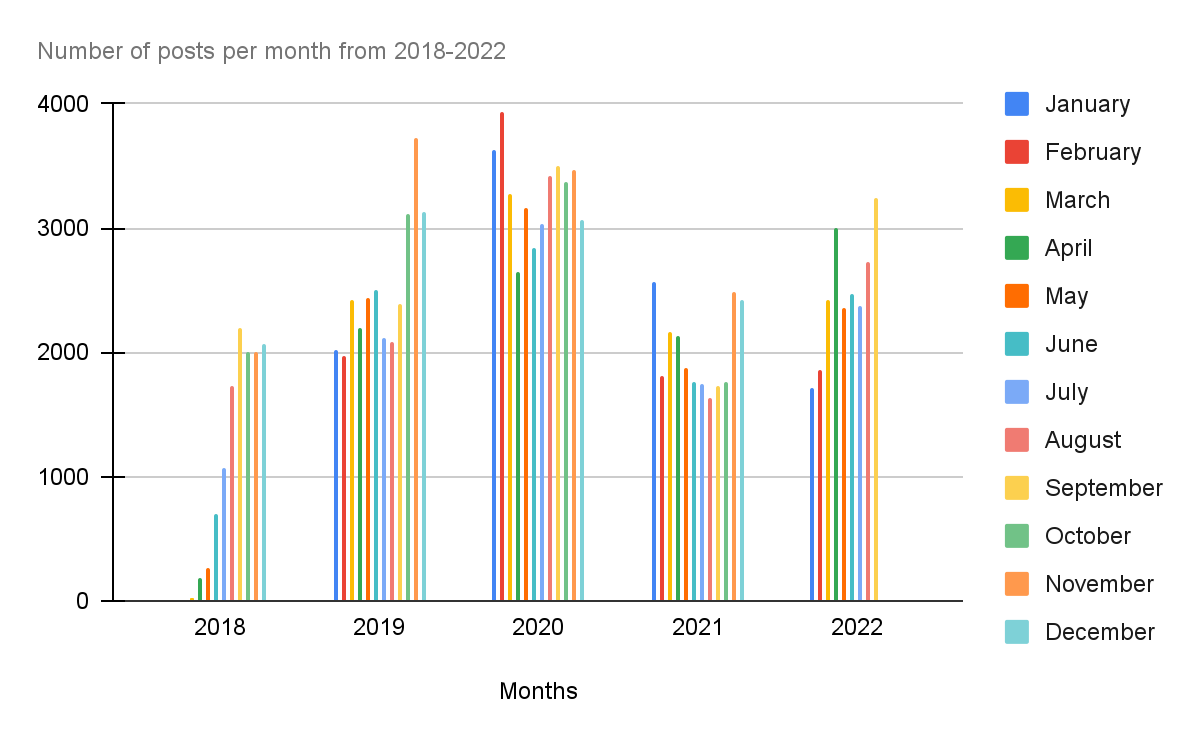


**Table S2.** Full list of sourcing-related keywords used.

| procure | procured | procures | source | sources | sourced |
| --- | --- | --- | --- | --- | --- |
| sells | sell | sold | seller | sellers | buy |
| buys | bought | get sn from | gets sn from | getting sn from | got sn from |
| purchase | purchases | purchased | order | orders | ordered |

**Figure S2.** Sodium nitrite-related topics: unigrams.


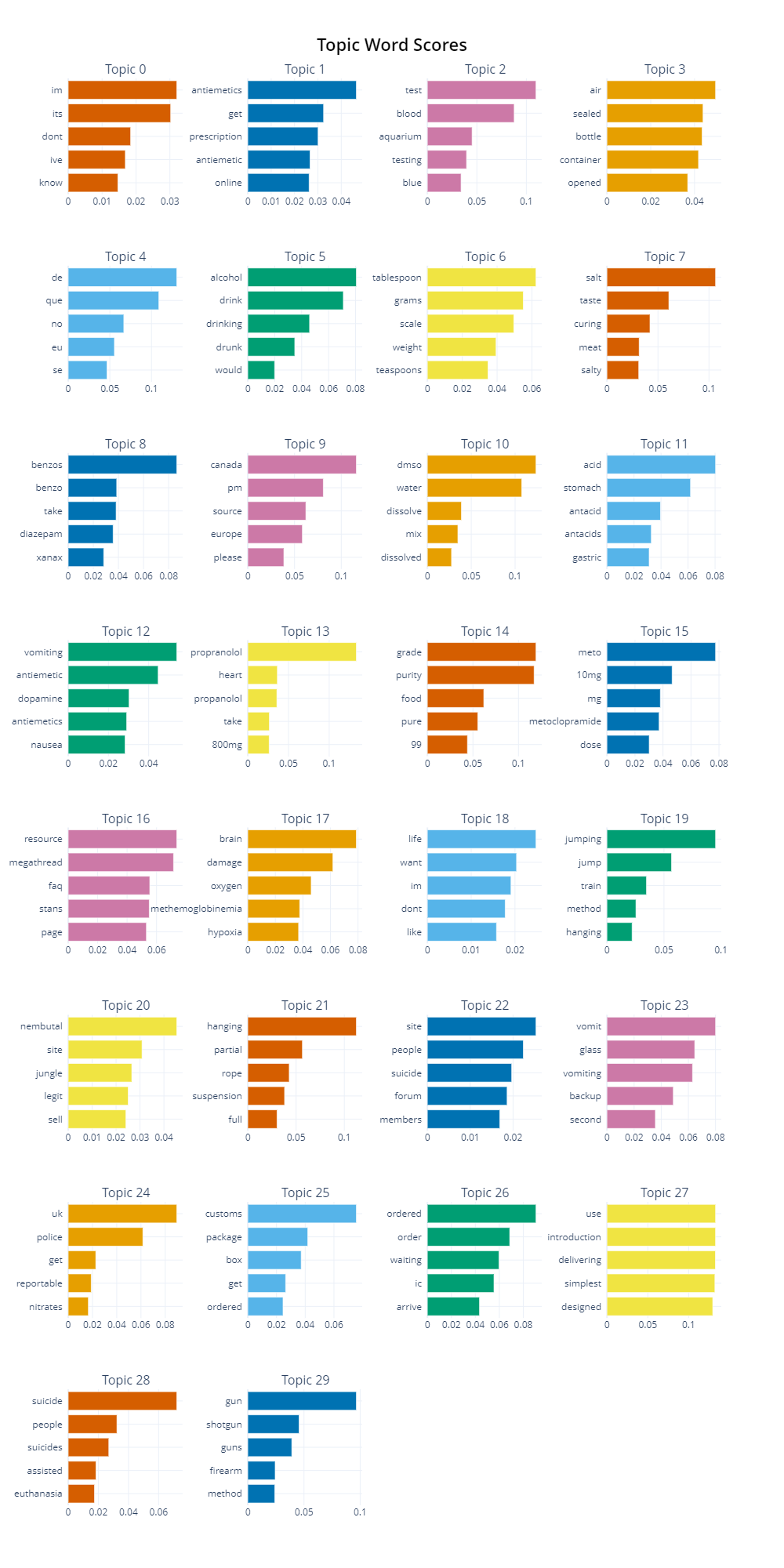


**Figure S3.** Sodium nitrite-related topics:
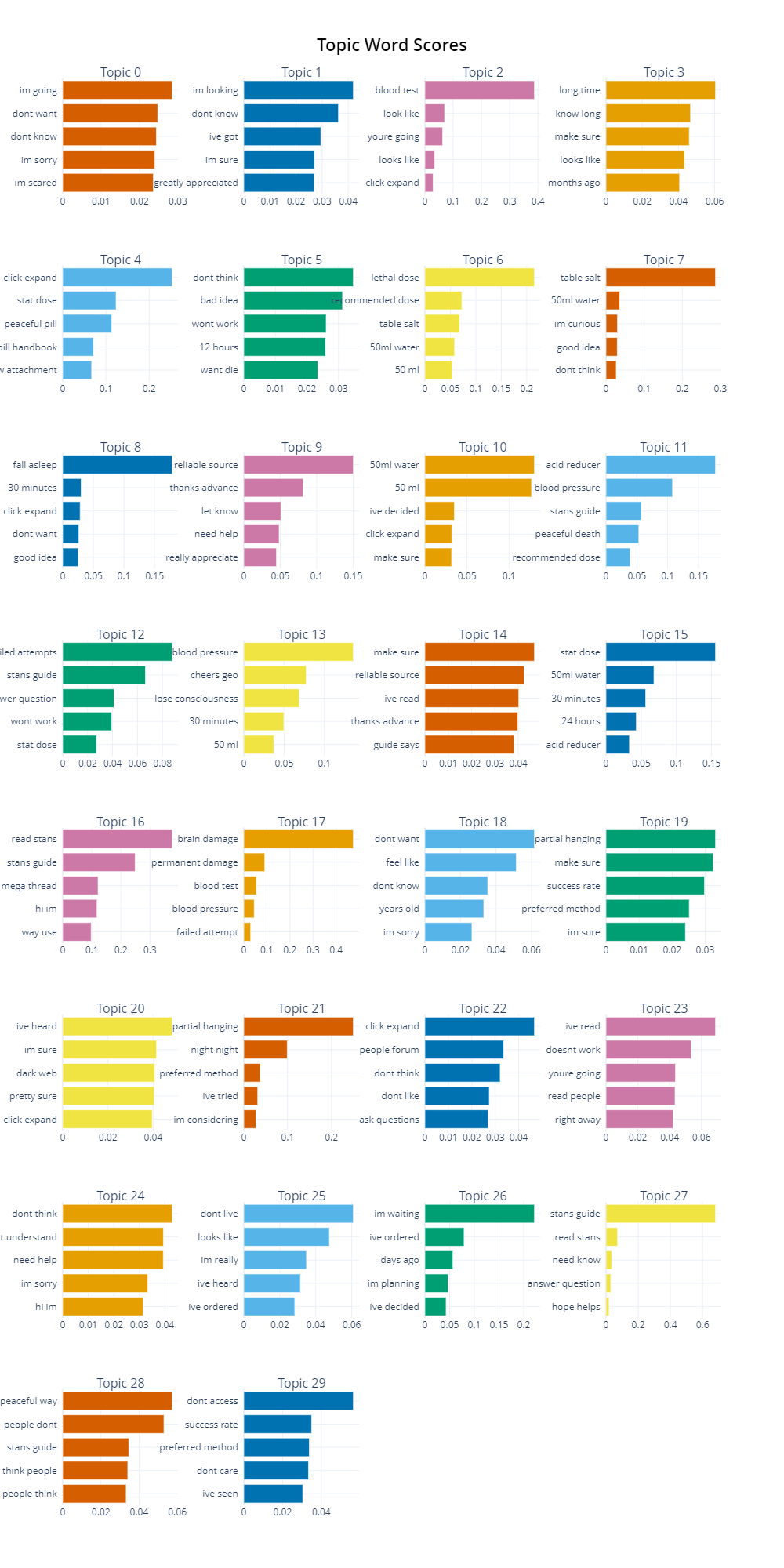
bigrams.

**Code snippet.** Rule-based pattern recognizer in spaCy

ruler = nlp.add_pipe(‘entity_ruler’)

pattern = [{‘label’: Suicide_Method’, ‘pattern’:’sn’}]

ruler.add_patterns(pattern)

**Code repository**

<https://github.com/das-sudeshna/sodium-nitrite>
